# Supplementary material for: Transcriptomics Reveal the Effects of Breeding Temperature on Growth and Metabolism in the Early Developmental Stage of Platax teira
Source: Biology (Basel). 2023 Aug 23;12(9):1161. doi: 10.3390/biology12091161 (PMC10525949; doi:10.3390/biology12091161)
Supplement: Supplementary file 1 [file biology-12-01161-s001.zip › biology-2437861-supplementary/supplementary data/Supplementary.pdf]

Supplementary Table S1 Disembarkation data

| Sample | Raw reads | Raw bases | Error rate (%) | Q20(%) | Q30(%) | GC content (%) |
|---|---|---|---|---|---|---|
| 21°C-1 | 47742756 | 7.21E+09 | 0.026 | 97.49 | 93.39 | 48.89 |
| 21°C-2 | 49561682 | 7.48E+09 | 0.0263 | 97.32 | 93.15 | 46.97 |
| 21°C-3 | 47584150 | 7.19E+09 | 0.0261 | 97.44 | 93.3 | 46.69 |
| 24°C-1 | 51560346 | 7.79E+09 | 0.0259 | 97.52 | 93.5 | 49.94 |
| 24°C-2 | 41554762 | 6.27E+09 | 0.0259 | 97.48 | 93.51 | 48.32 |
| 24°C-3 | 57901326 | 8.74E+09 | 0.0258 | 97.52 | 93.56 | 48.82 |
| 27°C-1 | 50249480 | 7.59E+09 | 0.0254 | 97.72 | 93.94 | 50.3 |
| 27°C-2 | 45434242 | 6.86E+09 | 0.026 | 97.43 | 93.42 | 47.47 |
| 27°C-3 | 46846462 | 7.07E+09 | 0.026 | 97.46 | 93.41 | 47.45 |
| 30°C-1 | 49649280 | 7.50E+09 | 0.0257 | 97.59 | 93.6 | 50.5 |
| 30°C-2 | 59157916 | 8.93E+09 | 0.0259 | 97.45 | 93.47 | 50.34 |
| 30°C-3 | 57766186 | 8.72E+09 | 0.0256 | 97.6 | 93.74 | 50.17 |

**Supplementary Table S2 Data filtering statistics**

| Sample | Clean reads | Mapped reads | Mapped ratio |
|---|---|---|---|
| 21°C-1 | 23682465 | 17891764 | 75.55% |
| 21°C-2 | 24541157 | 17927444 | 73.05% |
| 21°C-3 | 23592874 | 17034968 | 72.20% |
| 24°C-1 | 25562748 | 19796853 | 77.44% |
| 24°C-2 | 20576389 | 15797221 | 76.77% |
| 24°C-3 | 28669801 | 22291945 | 77.75% |
| 27°C-1 | 24937614 | 19177963 | 76.90% |
| 27°C-2 | 22472441 | 16995080 | 75.63% |
| 27°C-3 | 23195794 | 17425182 | 75.12% |
| 30°C-1 | 24624303 | 19035171 | 77.30% |
| 30°C-2 | 29293587 | 23244916 | 79.35% |
| 30°C-3 | 28624550 | 22801491 | 79.66% |
